# QM/MM free energy Simulations of an efficient Gluten Hydrolase (Kuma030) Implicate for a Reactant-State Based Protein-Design Strategy for General Acid/Base Catalysis

**DOI:** 10.1038/s41598-018-25471-z

**Published:** 2018-05-04

**Authors:** Xia Wang, Ruirui Li, Wenchao Cui, Qiang Li, Jianzhuang Yao

**Affiliations:** School of Biological Science and Technology, University of Jinan, Jinan, 250022 China

## Abstract

It is a grand attraction for contemporary biochemists to computationally design enzymes for novel chemical transformation or improved catalytic efficiency. Rosetta by Baker *et al*. is no doubt the leading software in the protein design society. Generally, optimization of the transition state (TS) is part of the Rosetta’s protocol to enhance the catalytic efficiency of target enzymes, since TS stabilization is the determining factor for catalytic efficiency based on the TS theory (TST). However, it is confusing that optimization of the reactant state (RS) also results in significant improvement of catalytic efficiency in some cases, such as design of gluten hydrolase (Kuma030). Therefore, it is interesting to uncover underlying reason why a better binding in the RS leading to an increased *k*_*cat*_. In this study, the combined quantum mechanical/molecular mechanical (QM/MM) molecular dynamics (MD) and free energy (PMF) simulations, p*K*_*a*_ calculation, and the statistical analysis such as the ANOVA test were carried out to shed light on the interesting but elusive question. By integration of our computational results and general acid/base theory, we answered the question why optimization of RS stabilization leads to a better TS stabilization in the general acid/base catalysis. In addition, a new and simplified protein-design strategy is proposed for the general acid/base catalysis. The idea, that application of traditional well-defined enzyme mechanism to protein design strategy, would be a great help for methodology development of protein design.

## Introduction

Enzymes are fundamental catalysts that are involved in most life processes^1,2^. enzymes are no doubt highly efficient catalysts, with significantly enhanced reaction rates compared to the corresponding uncatalyzed reactions in solution^3–5^. Therefore, it is of fundamental and practical importance to understand the origin of the detailed catalytic mechanisms of the enzymes. In order to explain the origin of the catalytic effect of enzymes, the uncatalyzed reaction in solution is generally used as a reference state. Thus, the catalytic effect (*k*_cat_/*k*_o_) is evaluated by the rate constant of *k*_cat_ for enzymatic reaction divided by the rate constant (*k*_o_) for the corresponding uncatalyzed reaction in water. The enhancement factor of the catalytic effect sometimes can achieve more than 10^17 ^^5^. The origin of the catalytic effect has been studied extensively and ideas have been proposed to explain the origin of the catalytic effect of the enzymes^6^. One general and traditional explanation proposed by Linus Pauling is the transition state theory (TST) in which the catalytic efficiency of enzyme is due to the better binding between enzyme and substrate in the transition state (TS) rather than in the reactant state (RS)^7^. In other words, enzyme and substrate binds tighter in TS rather than RS. Another basic idea related to the catalytic power of enzymes is that the functional groups of the catalytic residues are perfectly oriented^8^. Despite the rightness of this classical idea, it is still not clear how the stronger binding and perfect orientation achieved by enzymes in the transition state. To fill the gap, catalytic opinions are proposed continually. The important modern understanding of enzymatic catalytic efficiency is summarized here by terms, including electrostatic preorganization, near-attack conformer, proton tunneling, reactant destabilization, dynamical effects, acid/base catalysis, covalent catalysis, and so on.

Compared to the fruitful insights by understanding the enzyme mechanism, protein (enzyme) design seems still at its infancy, in the sense that it remains challenging to screen mutations by both experimental work and make quantitative predictions on the catalytic proficiency (*k*_cat_/*K*_M_) of an engineered enzyme with an entirely generic computational approach^9^. In recent decades, protein design is advanced in two directions, including *de novo* enzymes design (creation of new enzymes) and re-design of naturally-evolved enzymes to gain a significantly improved catalytic proficiency^10,11^. As is well-known, design methodology provided by Rosetta is the leading computational approach in the enzyme design society^12^. A couple of successful design cases had been shown by previous studies using Rossetta^10,11,13,14^. The design protocol available in Rosetta can be briefly summarized as below^12^. First, modeling the active site with minimal residues according to the chosen catalytic mechanism; second, searching the minimal active site in a scaffold protein database; third, enhancement of the transition state stabilization by re-design of the surrounding residues in the active site; fourth, proving and ranking the designed enzymes by experimental work. Before the optimization of the TS complex by mutation of the active site residues, the detailed catalytic mechanism of the enzymes must be understood clearly and the rate-limiting transition-state complex needs to be modeled firstly by the QM method. The whole process is still too complicated for non-computational biochemists, so a simplified procedure is expected and would be a great help for protein design society. The well-defined catalytic mechanisms may be a potential theory database for development of the easy-to-use protein design approach. Thereby, it is interesting to ask whether the theories of the catalytic mechanism of enzymes (e.g., general acid/base catalysis) discussed above can help to simplify the procedure of protein design and thus advance the progress of next level methodology development in enzyme design.

Generally speaking, all discovered naturally-evolved enzymes play their catalytic roles strictly following the well-known rules (as discussed above), such as enzymes in the Sedolisin family^15^. As described by our previous computational studies^16,17^, Sedolisins adopt (but not limited to) the acid/base and the covalent catalysis to perform their peptidase role. For example, the wild type kumamolisin-As (KumaWT) shares the similar catalytic mechanism with other Sedolisins^18^, but has some specific properties^19,20^. First, the optimal pH for enzymatic activity is pH2–4; Second, the high activity an stability at the 37 ^◦^C (the physiologic temperature); Third, KumaWT as a peptidase exhibits a substrate specificity for Pro at the P2 position; Fourth, a good amount of soluble protein is obtained by expression and purification of the recombinant KumaWT using a standard protocol in *E*. *coli*. These properties make KumaWT as an ideal enzyme scaffold to develop an oral enzyme therapeutic for celiac sprue caused by gluten in everyday food consuming^21^. The PQ motif found in the immunogenic peptides of gluten is proved to be the immunological antigens leading to a series of immune response (e.g., inflammatory reactions) in human the digestive tract^22^. However, the substrate specificity of KumaWT favors positive charged residues (e.g., His, Arg, and Lys) in the S1 binding pocket^20^. To change the substrate specificity of KumaWT toward PQ-containing peptides, Gordon *et al*. computationally designed 261 mutants by Rosetta, and named the most active design as Kuma010^21^. Mature Kuma010 owns six mutations: S73K, N102D, D104T, G130S, D169G, D179H. Experimental study proved that Kuma010 shows more than 100 fold better peptidases reactivity than KumaWT. Using Rosetta, Kuma010 was further re-designed by Wolf *et al*.^23^, in order to gain greater catalytic activity and broader substrate specificity. Compared to Kuma010, the new design named Kuma030 represents a 30-fold better catalytic efficiency. Seven mutations (K73E, E80T, S165Q, G169S, D210Q, A260Q, I274T) were introduced to Kuma030. The Kuma010 and Kuma030 design approach basically adopted the general design protocol of Rosetta with a confusing assumption that the better enzyme-substrate binding in the RS leads to a better stabilization in TS, and thus increases the *k*_*cat*_^21,23^. In other words, Kuma030 was not designed by optimization of the TS complex, but the RS complex. According to the TST, an improved *k*_cat_ value must root in a better binding in TS than RS. The change of the binding free energy in RS can be roughly used to evaluate the Michealis constant (*K*_M_), not the turnover number (*k*_*cat*_). So, a gap exists between TST and Kuma030-design protocol adopted by Rosetta. Considering the assumption of Rosetta works in the design of gluten hydrolases, this elusive question motivated us to uncover the underlying reason why a better binding in the RS leading to an increased *k*_*cat*_. To clarify this confusing and fill the gap, the detailed catalytic mechanism of KumaWT and Kuma030 must be firstly understood by calculating the free energy profile along the RC. On the basis of completely understanding the fundamental enzymatic reaction mechanism and the origin of the improved catalytic efficiency of the mutant, next generation of protein design strategy is expected to be proposed.

With these questions in mind, we performed free energy (PMF) simulations based on the combined quantum mechanical/molecular mechanical (QM/MM)^24^ molecular dynamics (MD), p*K*_*a*_ calculation, and the statistical analysis such as the ANOVA test. This computational study firstly uncovers the fundamental catalytic mechanism of the KumaWT towards the peptide (PFPQPQQPF), and then investigates the origin of the improved catalytic efficiency of Kuma030. Finally, a simple, fast, and reliable approach for enzyme design on general acid/base catalysis is proposed.

## Results

**Figure 1:**
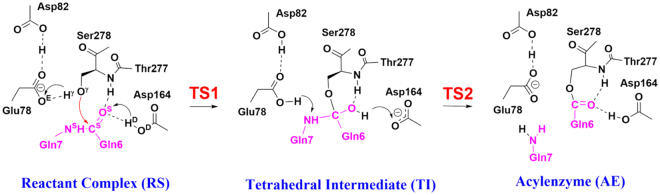
The proposed acylation catalytic mechanism of KumaWT, and Kuma030 complexed with a substrate peptide (PFPQPQQPF) and the role of important active-site residues. The reaction coordinates (RC) applied in this study are denoted as RC1 = r(O^γ^…H^γ^) − r(O^E^…H^γ^) − r(C^S^…O^γ^), RC2 = r(O^D^…H^D^) − r(O…H^D^), and RC3 = r(N^S^..C^S^) + r(O^E^…H^γ^) − r(N^S^…H^γ^). The RCs are all in Å.

The catalytic mechanism of the substrate (peptide PFPQPQQPF) hydrolysis by Kuma010 had been studied by our previous QM/MM study^25^, as shown in Fig. 1. Consistent with other Sedolisins and autocatalytic process of KumaWT, the reaction pathway consists of acylation and deacylation processes^16–18^. Consistent with the previous studies, the potential energy profile also shows the acylation process of Kuma030 with PFPQPQQPF is the rate-limiting step. Considering the catalytic turnover number (*k*_*cat*_) is related to the activation free energy barrier, only rate-limiting steps (acylation processes) were simulated by free energy simulations for both enzymes (KumaWT and Kuma030) toward peptide (PFPQPQQPF).

### Enzymes-PFPQPQQPF binding mode

**Figure 2:**
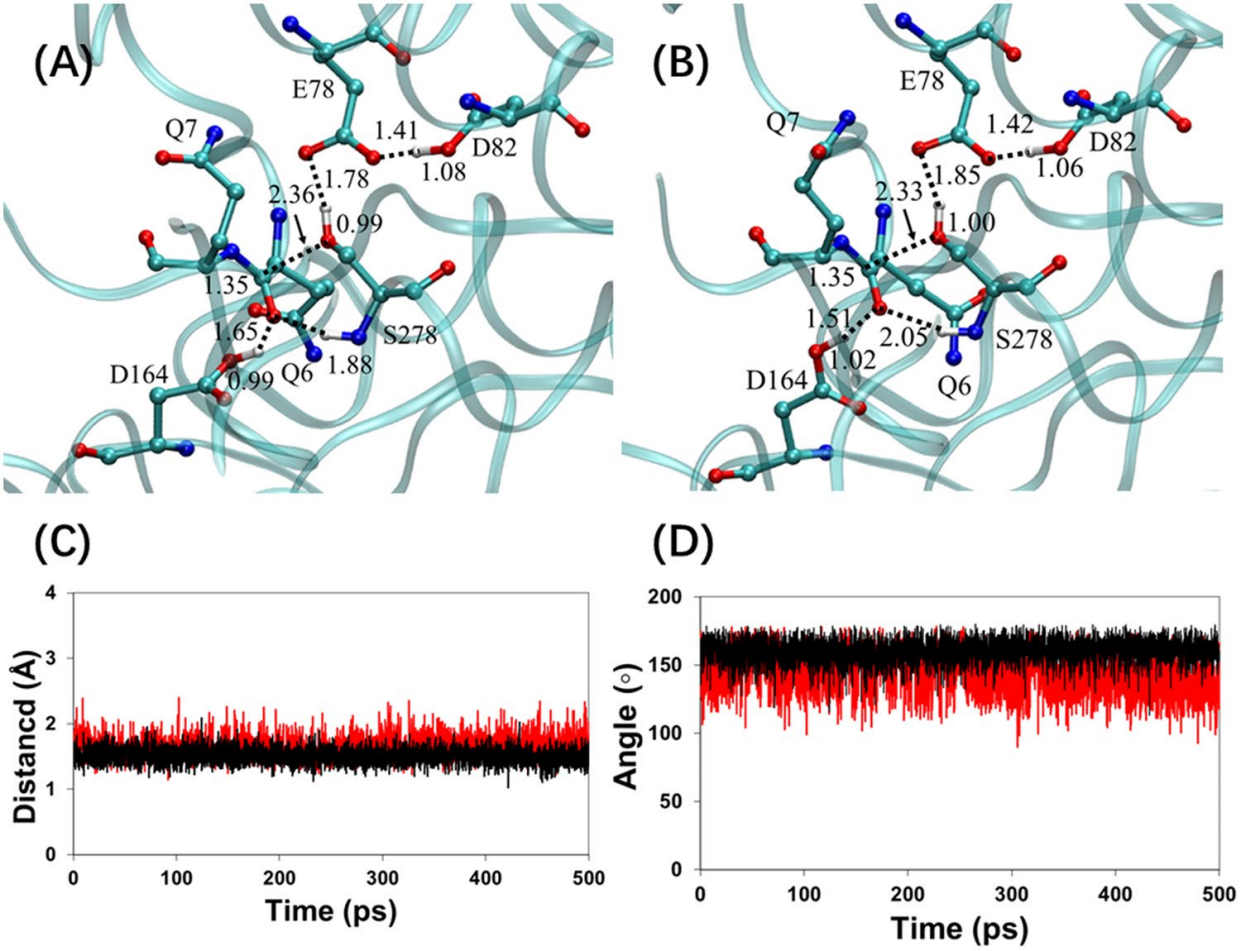
Reactant state (RS) complexes and distributions of distance and angle obtained by the QM/MM(DFTB3/CHARMM36) MD simulation. (**A**) Average structure of kumamolisin-As RS complex generated by 500 ps QM/MM MD simulation. (**B**) Average structure of Kuma030 RS complex generated by 500 ps QM/MM MD simulation. (**C**) Distance distribution of the hydrogen bond r(O^S^…H^D^) during 500 ps MD simulation. (**D**) Distribution of the hydrogen bond angle a(O^S^…H^D^-O^D^) during 500 ps MD simulation.

The QM/MM(DFTB3/CHARMM36) MD simulations, in which the atoms included in QM region were described by the third-order self-consistent charge density functional tight-binding (DFTB3, 3ob-2-1)^26^ method and the MM atoms were described by the classical CHARMM36 force field^27^, were performed to reveal the binding properties in the RS complexed formed by KumaWT and Kuma030 with the substrate (PFPQPQQPF). As shown in Fig. 2, average structures of active sites show a good interaction between substrates and the active sites of KumaWT and Kuma030. The important residues of KumaWT and Kuma030 consist of the catalytic triad (E78, D82, and S278) and residues forming the oxyanion hole. The oxyanion hole is constructed by the side chain of Asp164 and the amide groups of Ser278. The hydrogen bond networks were formed within the catalytic triad in both RS complexes. The hydrogen bond network is formed among the catalytic triad^16–18^. KumaWT and Kuma030 share the same patterns on the interactions between enzymes and substrates in the active sites, but the strength of the interactions is slightly different with each other. On one hand, the Ser278 O^γ^ atoms of KumaWT and Kuma030 interact well with the P1-Gln6 C^S^ of substrate (PFPQPQQPF). The corresponding interaction distances are 2.36 and 2.33 Å, respectively. Those distances make the O^γ^ atoms of Ser278 in good position for nucleophilic attack toward the C^S^ atoms of the substrates. On the other hand, the carbonyl O^S^ atom of the P1-Gln6 backbone forms two hydrogen bonds in the oxyanion hole, which are donated by the protonated side chain of Asp164 and the NH group of Ser278 backbone. The Asp164 in Kuma010 and corresponding Asp170 in Sedolisins are suggested to function as general acid/base catalysts in the acylation processes^15,28^. Interestingly, the average hydrogen-bond distances are 1.65 and 1.51 Å for RS complexes of KumaWT and Kuma030, respectively. The stronger hydrogen bond of Kuma030 may partly account for the better binding designed by Rosetta in the RS compared to KumaWT.

### Acylation reaction pathway in KumaWT and substrate complex

**Figure 3:**
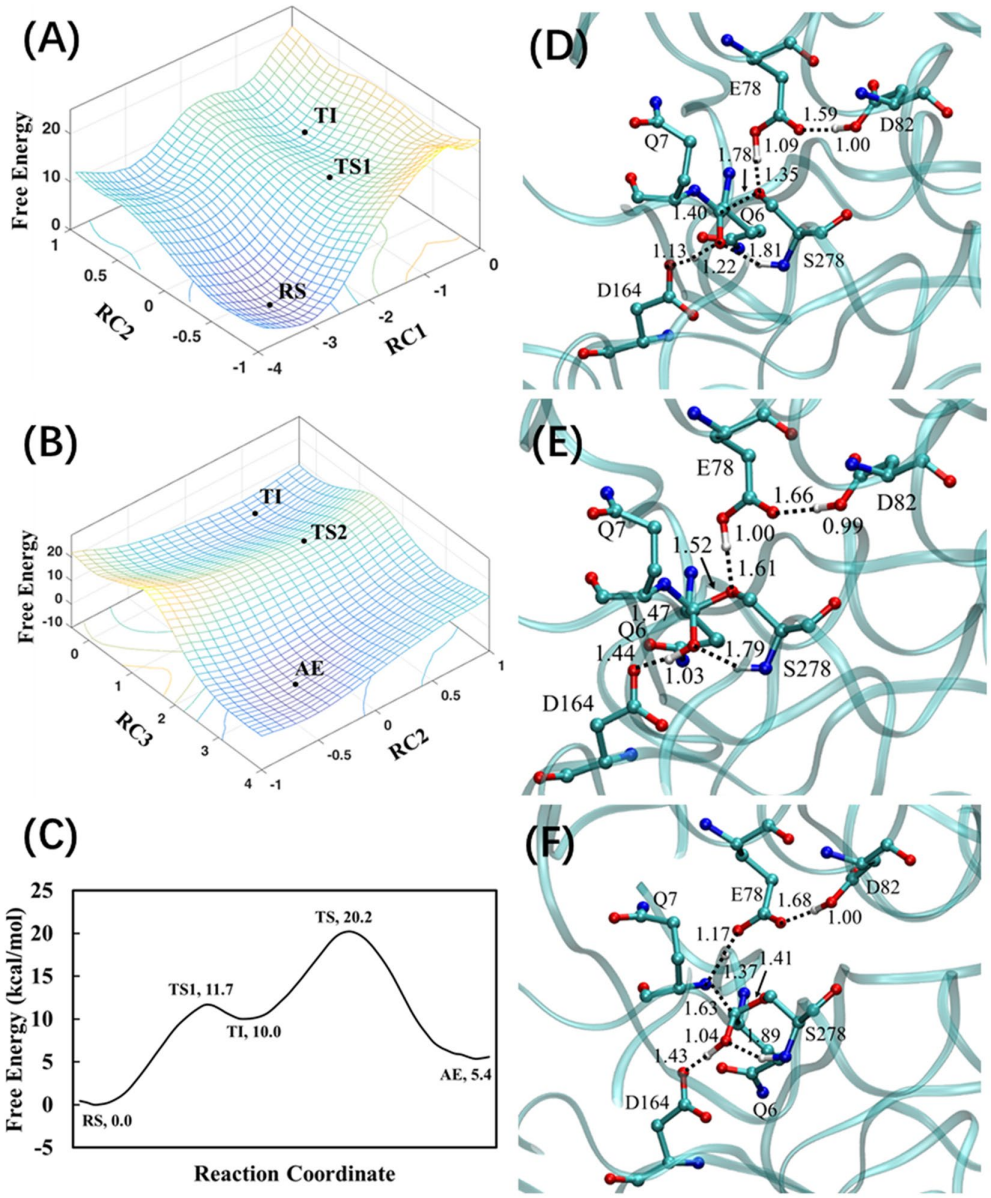
Free energy maps and average structures of KumaWT with the substrate peptide (PFPQPQQPF) for acylation process. (**A**) Use of RC1 and RC2 to simulate the formation of the TI in the acylation process; (**B**) RC2 and RC3 to simulate the decomposition of the TI. (**C**) Minimum free energy profile plotted on the basis of Fig. 3A,B. Average structures of TS1 (**D**), TI (**E**), and AE (**F**). The distances are all in Å. The energies are in kcal/mol. All transition states are approximate TS.

Using the KumaWT-PFPQPQQPF structure as the RS (Michaelis-Menten) complex (depicted in Fig. 2A), the hybrid QM/MM(DFTB3/CHARMM36) PMF (free energy) simulations were performed to simulate the acylation process of KumaWT toward the peptide (PFPQPQQPF). With two-sets RCs, the two-dimensional (2D) free energy (PMF) profiles were determined and plotted in Fig. 3A,B. Figure 3C shows the one dimensional minimum free energy profile generated based on Fig. 3A,B. All free energy profiles demonstrate that the RS complex went through two TSs and one tetrahedral intermediate (TI). Acyl-enzyme (AE) was finally formed at the end of the acylation process of KumaWT. The whole acylation process follows the classical catalytic mechanism of the Sedolisins family^15,25^.

As shown in Fig. 3D–F, the acylation process includes TS1, TI, TS2, and AE stages. The acylation process consists of formation of a C^S^−O^γ^ bond between the P1-Gln6 backbone of substrate and the Ser278 side chain of KumaWT (related to TS1) and breaking of the C^S^−N^S^ peptide bond of the substrate (related to TS2). Simultaneously, two protons are transferred associated with these two TSs. In the TS1, the H^γ^ atom located at Ser278 sidechain is transferred to Glu78 O^E^ atom, while the H^D^ atom located at Asp164 is transferred to the P1-Gln6 backbone O^S^ atom. In the rate limiting TS2, the H^γ^ atom of Ser278 currently located at the O^E^ atom of the Glu78 side chain is transferred to the backbone N^S^ atom of the P2-Gln7, while the H^D^ atom currently located at the P1-Gln6 backbone O^S^ atom is transferred back to carboxylate group of Asp164 sidechain.

As shown in Fig. 3, the chosen reaction coordinates are similar with previous Kuma010 QM/MM study^25^. For instances, RC1 = r(O^γ^…H^γ^) − r(O^E^…H^γ^) − r(C^S^…O^γ^), RC2 = r(O^D^…H^D^) − r(O^S^…H^D^), and RC3 = r(N…C) + r(O^E^…H^γ^) − r(N…H^γ^) and RC2.

### Acylation reaction pathway in Kuma030 and substrate complex

**Figure 4:**
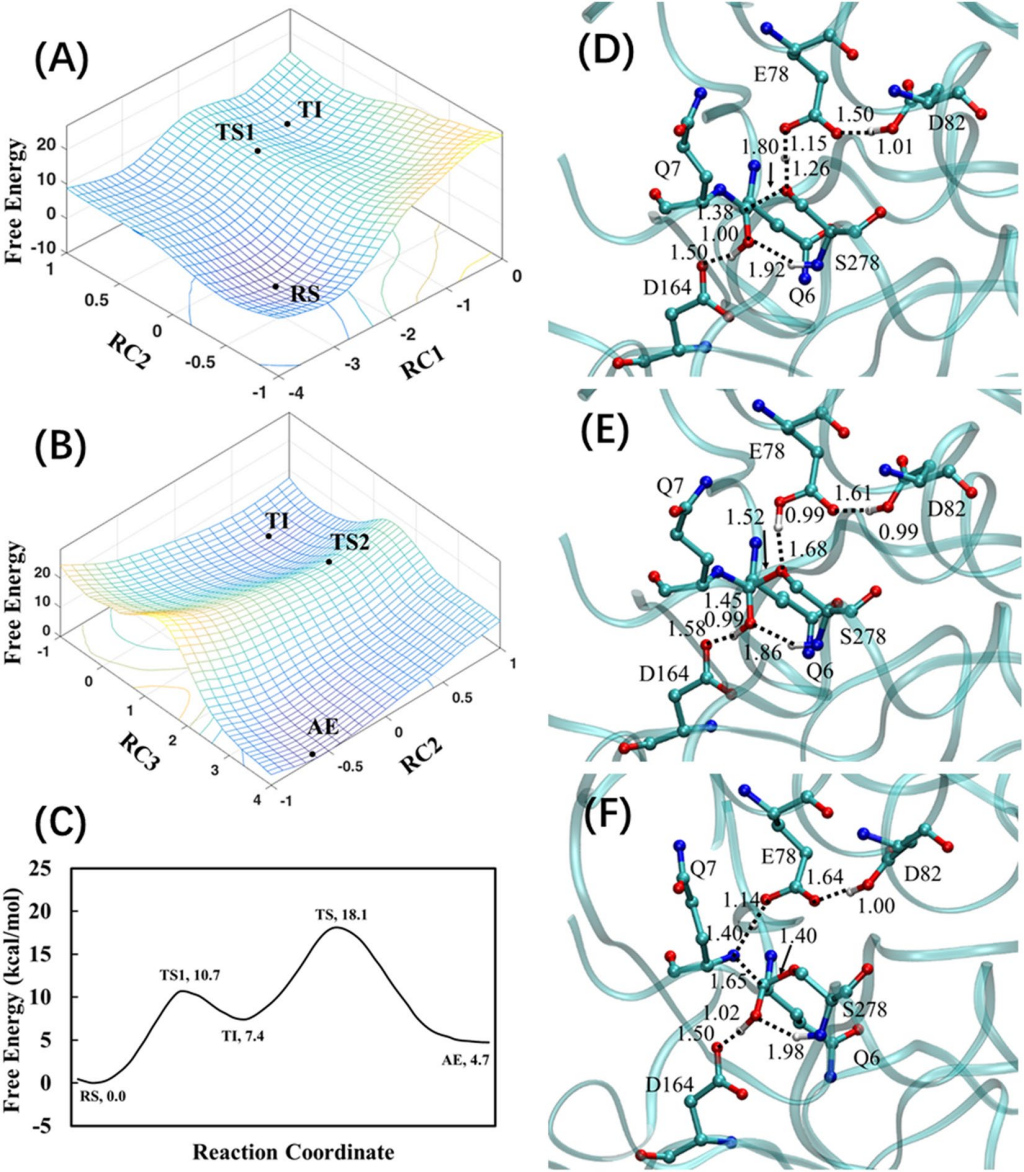
Free energy maps and average structures of Kuma030 with the substrate peptide (PFPQPQQPF) for acylation process. (**A**) Use of RC1 and RC2 to simulate the formation of the TI in the acylation process; (**B**) RC2 and RC3 to simulate the decomposition of the TI. (**C**) Minimum free energy profile plotted on the basis of Fig. 4A,B. Average structures of TS1 (**D**), TI (**E**), and AE (**F**). The distances are all in Å. The energies are in kcal/mol. All transition states are approximate TS.

Using RS complex as the starting point, the same QM/MM(DFTB3/CHARMM36) PMF simulations were carried out to simulate the acylation process of Kuma030 with the substrate peptide (PFPQPQQPF), as shown in Fig. 4A,B. The minimum free energy profile (depicted in Fig. 4C) demonstrates that Kuma030 shares the similar acylation mechanism with KumaWT, including a TI sandwiched by two TSs. Therefore, the two sets of RCs are chosen identically with KumaWT simulations.

Figures 4D–F show the two-steps chemical transformation from the reactant state (RS) to the tetrahedral intermediate (TI) through transition state (TS1), and from TI to acyl-enzyme (AE) through the transition state (TS2). In the TS1 stage of Kuma030, the H^D^ atom of Asp164 has been transferred to the P1-Gln6 O^S^ atom with a distance r(O^S^…H^D^) of 1.00 Å (see Fig. 4D), while the corresponding proton transfer process in KumaWT is still at the middle stage with the r(O^S^…H^D^) = 1.22 Å (see Fig. 3D). The similar trend was also found in the H^γ^ atom proton transfer process. For example, the distances of r(O^E^…H^γ^) are 1.35 and 1.26 Å, respectively. In the TI as shown in Figs 3E and 4E, all of the H^γ^ atom of KumaWT and Kuma030 have been transferred to the Glu78 O^E^ atom. Similarly, all of the H^D^ atom of KumaWT and Kuma030 have been transferred to the O^S^ atom of the P1-Gln6 with the distances of 1.03 and 0.99 Å, respectively. In the TS2 as shown in Figs 3F and 4F, a similar proton transfer pattern was found between KumaWT and Kuma030. For instance, the transfers of the H^γ^ atom to the N^S^ are all in their middle stage with distances r(N…H^γ^) of 1.37 and 1.40 Å, respectively for KumaWT and Kuma030, while the transfers of the H^D^ atom back to Asp164 are all in its beginning stage with the corresponding distances r(N…H^γ^) of 1.43 and 1.50 Å.

### Calculated free energy profiles and experimental kinetic data

According to the minimum free energy profiles depicted in Figs 3C and 4C, both acylation processes of KumaWT and Kuma030 are two steps reactions involving a RS, two TSs, a TI, and an AE. As discussed above, the acylation is the rate limiting step for the KumaWT and Kuma030 toward the substrate peptide (PFPQPQQPF), so the activation free energy barriers can be read from the minimum free energy profiles. The free energy barriers (corresponding to TS1 and TS2) of Kuma030 are 10.7 and 18.1 kcal/mol, respectively. So, the rate-limiting step is associated with the second TS in the acylation process of Kuma030, which is consistent with the wild type Sedolisins and Kuma010 catalyzed reaction. The *k*_*cat*_ value is not available for KumaWT with the substrate peptide (PFPQPQQPF), while the *k*_*cat*_ value for Kuma030 can be calculated based on the experimental Michaelis-Menten curves^23^. As shown in the Figure S1 of the Kuma030 experimental study^23^, the *k*_*cat*_ value for Kuma030 is roughly 75.7 S^−1^. According to the conventional TST^29^, the experimental derived activation free energy barrier (under 37 °C) is15.5 kcal/mol. Our previous benchmark calculation for Kuma010 with the substrate peptide (PFPQPQQPF) showed that DFTB3/MM overestimates the potential energy barrier by around 3.1 kcal/mol in the rate-limiting TS, compared to B3LYP/MM method^30^. After an energy correction of DFTB3/MM systematical error, the calculated activation free energy barrier of Kuma030 with the substrate peptide (PFPQPQQPF) is 15.1 kcal/mol, which is in a good agreement with the experimental result (15.5 kcal/mol).

## Discussion

Although numerous chemical transformations had been found and characterized in the naturally evolved enzymes, it is still super attractive for computational biochemists to design proteins with novel or improved catalytic functions. The major challenge for modern protein design is to develop efficient methods for ranking of designed enzymes before experimental proof^12^.

Computational ranking of the *k*_*cat*_ is possible, because the enzymatic reaction rates (*k*_*cat*_) and the corresponding activation free energy barrier can be accurately converted based on the TST^31^. To computationally determine the value of *k*_*cat*_ for the chemical steps catalyzed by native or engineered enzymes, the corresponding activation free energy barrier can be obtained based on the calculations of the free energy profiles along the reaction coordinate (RC). The combined QM/MM approaches^24^, are the choice to compute the free energy difference along the RC by potential of mean force (PMF) simulations and thus determine the activation free energy barriers for enzyme-catalyzed reactions. QM/MM methods based on high level QM basis set are still too computationally expensive to perform thousands of QM/MM simulations simultaneously, which is required by mutates screening of enzyme design protocol of Rosetta. Alternatively, semiempirical QM approaches, such as the self-consistent-charge density-functional-tight-binding (SCC-DFTB), have been developed^32,33^ to expedite the QM/MM MD simulations. However, supercomputers with hundreds of the processers are still needed to calculate thousands of free energy profiles for the mutants designed by Rosetta. Although possible, it is still a tremendous burden for experimental labs and even computational labs. To this end, it is an urgent but challenging work to develop simple, fast, and reliable method(s) to rank the *k*_*cat*_ values of designed enzymes before the experimental work. In this study, we tried to build up efficient ranking method by examination the well-known catalytic mechanism of enzymes, such as general acid/base catalysis.

General acid/base catalysis is the fundamentally important catalytic mechanism widely adopted by naturally-evolved enzymes, such as serine proteinases^34,35^. Acid/base catalysis improves the catalytic activity by proton-transfer process. In the rate limiting step of enzyme-catalyzed reactions, a proton can be transferred either to substrates (reactant) from active site acid residues or from substrates to active site base residues, which results in reducing the activation free energy barriers. Interestingly, the activation free energy barrier of the proton transfer reaction can be lowered by reducing the p*K*_*a*_ difference between proton donor and acceptor^36,37^. Under the matching p*K*_*a*_ condition, the coupling of proton transfer can be maximized, leading to the shortening of the length of the hydrogen bond^37–39^. The length of the hydrogen bond can be analyzed by the strength and thus angle of the hydrogen bond. Thus, *k*_*cat*_ changes of mutants can be roughly measured by p*K*_*a*_ difference between proton donor and acceptor for proton-transfer reaction involved in the rate-limiting step. Because length and strength of the hydrogen bond is coupled with the p*K*_*a*_ difference^37–39^, it is reasonable to propose that the *k*_*cat*_ changes may also be ranked by comparison of p*K*_*a*_ difference, length and angle of hydrogen bonds in the RS.

In this study, we use KumaWT and Kuma030 with higher *k*_*cat*_ designed by Rosetta as an example to test our hypothesis. First, the relationship between p*K*_*a*_ difference and corresponding free energy barrier was studied. In the first step of acylation process, the TS1 of Kuma030 shows a lower free energy barrier (10.7 kcal/mol) compared to KumaWT (11.7 kcal/mol). The proton transfer from the Asp164 to the P1-Gln6 is involved in the TS1. To measure the p*K*_*a*_ difference in the RS, we assume that the p*K*_*a*_ change of the O^S^ atom of the P1-Gln6 can be negligible upon the mutation of KumaWT to Kuma030 and the p*K*_*a*_ change of the O^S^ atom of the P1-Gln6 along the RC due to the developing charge increase is the same between KumaWT and Kuma030. So, the change of p*K*_*a*_ difference from KumaWT to Kuma030 can be determined by the p*K*_*a*_ change of the O^D^ atom of Asp164. The optimized RS complexes from the last snapshots of the MD simulation for both KumaWT and Kuma030 were submitted to H ++ using default setting (pH 4.0)^40^. The predicted p*K*_*a*_ of KumaWT is 6.30, while the predicted p*K*_*a*_ of Kuma030 is 5.86, which implicate for a reducing p*K*_*a*_ difference from KumaWT to Kuma030, thus resulting in a decreased TS1 free energy barrier of Kuma030 compared to KumaWT. The result is consistent with our PMF calculation, suggesting that a simple p*K*_*a*_ calculation is good enough for the *k*_*cat*_ ranking purpose in the general acid/base catalysis.

**Figure 5:**
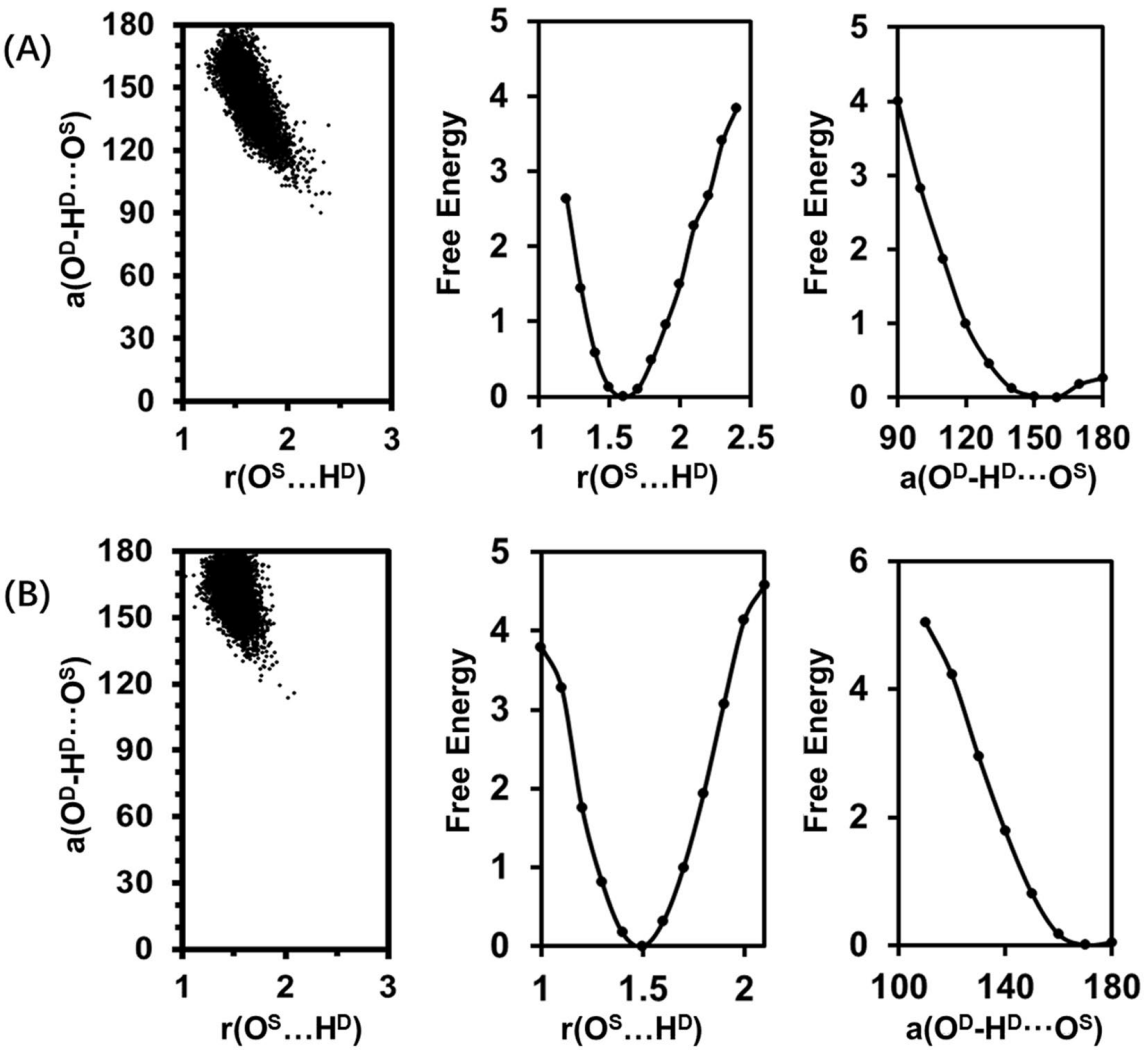
The distributions of r(O^S^…H^D^) and a(O^S^…H^D^-O^D^) and the corresponding free-energy changes from the 500 ps QM/MM MD simulations on the reactant complexes of kumamolisn-As (**A**) and Kuma030 (**B**). (*Left*) The two-dimensional plot of r(O^S^…H^D^) and a(O^S^…H^D^-O^D^) distributions. (*Center*) The free-energy change as a function of r(O^S^…H^D^) obtained from the distributions. (*Right*) The free-energy change as a function of a(O^S^…H^D^-O^D^) obtained from the distributions. The distances are given in Å. The angles are in degree (°). The energies are in kcal/mol.

Second, to test our hypothesis that the free energy barriers can be ranked by comparison of the hydrogen bond lengths in the RSs, the relationship between *k*_*cat*_ and bond lengths and angles is investigated by analyses of the distributions of the hydrogen bond length r(O^S^…H^D^) and angle a(O^S^…H^D^-O^D^) as well as the corresponding free energy changes, as shown in Fig. 5. Compared to KumaWT, the Kuma030 shows a large population for the structures of hydrogen bond with relatively short r(O^S^…H^D^) and large a(O^S^…H^D^-O^D^), which is consistent with the average distances and angles depicted in Fig. 2. In the wise of free energy changes along with r(O^S^…H^D^) and a(O^S^…H^D^-O^D^), Kuma030 shows relatively low free energies at short r(O^S^…H^D^) and large a(O^S^…H^D^-O^D^), and implicates for an easy proton transfer process in term of the free energy barrier, which agrees well with the free profiles of the first proton transfer reactions between Asp164 and P1-Gln6 obtained by PMF calculations (see Figs 3C and 4C). Indeed, this is also the case for the second proton transfer processes between Asp164 and P1-Gln6 with a reverse result. In the TIs depicted in Figs 3E and 4E, KumaWT shows a better r(O^D^…H^D^) with a distance of 1.44 Å compared to the Kuma030 corresponding bond length with a distance 1.58 Å, suggesting that the free energy barrier of KumaWT for the second proton transfer process would be lower than Kuma030. Indeed, the free energy barriers between TI and AE are 10.1 and 10.7 kcal/mol, respectively. Our PMF results again support our hypothesis that short length of hydrogen bond leads to low free energy barrier of proton transfer process. Therefore, *k*_*cat*_ ranking task for general acid/base may be simply be done by comparing the corresponding hydrogen bond length without knowing the activation free energy barrier by performing expensive QM/MM simulations. Furthermore, the hydrogen bond lengths are 1.66 and 1.50 Å for the last snapshots minimized RS complex of KumaWT and Kuma030, respectively. Therefore, it is reasonable to propose that optimized hydrogen bond lengths can be used for the ranking purpose as well as the average distances.

**Figure 6:**
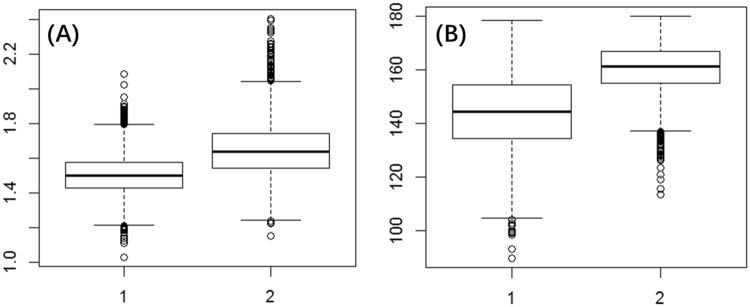
The one-way ANOVA test between RS complexes of kumamolisin-As and Kuma030. (**A**) distance r(O^S^…H^D^) comparison; (**B**) a(O^S^…H^D^-O^D^) comparison. Sample 1 is Kuma030 and Sample 2 is kumamolisin-As. 5000 windows were obtained based on 500 ps QM/MM MD simulation.

Finally, although bond length analysis seems as an easy way for the *k*_*cat*_ ranking task, we need to confirm that the difference of average bond lengths is statistically meaningful before the experimental work. In the RSs of KumaWT and Kuma030, the average bond lengths of r(O^S^…H^D^) are 1.65 and 1.51 Å, respectively. Since the bond lengths of r(O^S^…H^D^) are averaged from 5000 snapshots during the 500 ps MD simulation, we originally have 5000 data for each r(O^S^…H^D^) from KumaWT and Kuma030. The available data from MD simulations were further analyzed by the one-way ANOVA test implanted in R package^41^. ANOVA shows that distance and angle are significantly different between KumaWT and Kuma030, as shown in Table 1 and Fig. 6. Here, we propose for the first time the *k*_*cat*_ values of the general acid/base catalysis of designed enzymes may be ranked by comparison of the corresponding hydrogen bond length with the ANOVA test.

**Table 1:** One-way ANOVA test result.

data	Source of Variation	SS	df	MS	F	P-value
DIS	Between Groups	53.13601861	1	53.13602	2827.083	< 2e-16***
	Within Groups	187.9159414	9998	0.018795		
ANG	Between Groups	664067.812	1	664067.812	4707.510	< 2e-16***
	Within Groups	1410373.927	9998	141.066		

DIS: r(O^S^…H^D^); ANG: a(O^S^…H^D^-O^D^).

Computational protein design has been emerging as a leading and important research area in the biophysics/biochemistry. In this study, with the help of well-defined idea of the general acid/base catalysis, we firstly demonstrate that the ranking task for designed enzymes can be fulfilled not only by QM/MM studies, but also can be easily performed by comparison of the difference of p*K*_*a*_ or the bond lengths for the corresponding proton acceptor or donor in the general acid/base catalysis. For the proteins with the general acid/base catalysis as the rate-limiting step, the simplified protein-design protocol can be summarized as below. First, modeling the reactant state of the enzyme-substrate complex; second, mutants screening and equilibration and/or optimization of the RS complex by MD simulation and/or energy minimization; third, ranking the enhancement of the TS stabilization (*k*_cat_) by comparison of the hydrogen bond lengths involved in rate-limiting proton transfer step; fourth and optionally, ranking the enhancement of the binding free energy (*K*m) between substrate and protein; finally, proving the design experimentally. As a simple, fast, and reliable approach, it is expected to be a great help for protein design not only by Rosetta but also by the classical MD simulation and energy minimization.

## Methods

### Model construction

The binding model of Kuma030 and substrate (PFPQPQQPF) were built similarly with the Kuma030-design protocol described by Wolf *et al*.^23^. Briefly, the Kuma030-PFPQPQQPF complex were manually mutated based on our previously-simulated reactant state complex of Kuma010-substrate (PFPQPQQPF)^25^. Thus, six mutations (K73E/E80T/S165Q/G169S/D210Q/A260Q/) were introduced to Kuma010 in order to build Kuma030. The model of wild type kumamolisin As-substrate complex were from our previously study^25^.

Hydrogen atoms of enzyme-substrate complexes were added by the HBUILD module^42^ of CHARMM^43^. The protonation states of acidic and basic residues were determined under pH 4.0 condition depending on surrounding environment. All protonation states are confirmed by H++^40^. Using the O^γ^ atom of Ser278 as the enzyme-substrate complex center, the solvation of the system was performed with a 22 Å radius water droplet. Solvent water molecules close to crystal atoms (within 2.8 Å) were removed. The TIP3P water model^27,44–46^ was applied. The QM treated atoms include the side chains of E78, D82, D164, and S287, the carbonyl of substrate P5, the backbone of substrate Q6, and the C^α^ and amide group of substrate Q7. The MM treated atoms are the other atoms of the systems excluding the QM region. The QM and MM boundaries were treated by the divided frontier charge (*DIV*) link-atom scheme^43,47,48^. The SCC-DFTB module implemented in the CHARMM^49^ was employed for the QM-region atoms and the all-hydrogen CHARMM36 force field^27,44^ was employed for the MM atoms. The cut-off of the non-bonded interaction was 13 Å.

For the stochastic boundary^50^ MD simulation, the reference center is the side chain O^γ^ atom of Ser278. The radius (*r*) of the reaction region was of 20 Å, while the radius of buffer region was within 20 Å ≤ *r* ≤ 22 Å. Specifically, the reaction region was simulated using Newtonian equations-of-motion, while the buffer region was treated by solving Langevin equations-of-motion with a 298.15 K temperature bath of Langevin thermostat^51^. All atoms neither included in the reaction region nor buffer region were fixed. All hydrogen-involved covalent bonds were treated by the SHAKE algorithm^52^. The initial structures of the enzyme-substrate complexes were minimized firstly by the steepest descent (SD) method and then by the adopted-basis Newton-Raphson (ABNR) methods. A 1 femtosecond (fs) time step was set up for the following MD simulations. Starting from 50 K, the systems were heated to 298.15 K in 100 picosecond (ps) gradually and 500 picosecond MD simulations (production runs) were carried out for reactant state complexes (RS) of all enzyme-substrate complexes.

### The hydrogen bond length r(O^S^…H^D^) and angle a(O^S^…H^D^-O^D^) and the free energy change

The methodology was followed the previous protocol^53–55^. Briefly, based on the QM/MM MD simulationsof KumaWT and Kuma030, the distributions of r(O^S^…H^D^) and a(O^S^…H^D^-O^D^) were monitored during the 500 ps MD simulations for the RS complexes and applied to calculate the free energies required to generate the reactive structure. A one-way ANOVA on means was conducted in R package^41^ using the function *aov*.*out* to see if the important hydrogen bond related distances and angles are significantly different between KumaWT and Kuma030.

### The free energy (Potential mean force, PMF) simulation

The free energy (PMF) simulations were performed on the basis of the QM/MM(DFTB3/CHARMM36) MD simulations. The umbrella sampling method^56^ and the Weighted Histogram Analysis Method^57^ were employed to calculate the PMF profile as a function of the RCs. As the first step of free energy profiles determination for the acylation processes, the potential energy maps were determined by adiabatic-mapping calculations starting from the last snapshot of the 500 ps QM/MM MD simulations on the reaction systems. To obtain the 2D free energy maps, around 2000 windows were generated for each enzyme complex. For one window, 100 ps QM/MM MD simulation was carried out with the first 50 ps for equilibration. One snapshot was saved per 0.5 ps, so one hundred snapshots were obtained for one window. The force constant applied for the harmonic biasing potential was 150 kcal mol^−1^ Å^−2^ for all of the PMF calculations.

## Electronic supplementary material

Supplementary Information
