# Supplementary material for: QM/MM free energy Simulations of an efficient Gluten Hydrolase (Kuma030) Implicate for a Reactant-State Based Protein-Design Strategy for General Acid/Base Catalysis
Source: Sci Rep. 2018 May 4;8:7042. doi: 10.1038/s41598-018-25471-z (PMC5935664; doi:10.1038/s41598-018-25471-z)
Supplement: Supplementary file 1 — Supplementary Information [file 41598_2018_25471_MOESM1_ESM.docx]

**Supporting Information**

**QM/MM free energy Simulations of a Most Powerful Gluten Hydrolase (Kuma030) Implicate for a Reactant-State Based Protein-Design Strategy for the General Acid/Base Catalysis**

Xia Wang,^a^ Ruirui Li,^a^ Wenchao Cui,^a^ Qiang Li,^a^ and Jianzhuang Yao^a^*

*^a^*School of Biological Science and Technology, University of Jinan, Jinan 250022, China

**Correspondence to**:

Jianzhuang Yao, Ph.D.

Assistant Professor, School of Biological Science and Technology

University of Jinan

Jinan 250022, P.R. China

Phone: +86-531-82769122

E-mail: bio_yaojz@ujn.edu.cn

**Supporting Information Available:** Figure S1

**Additional Figures**


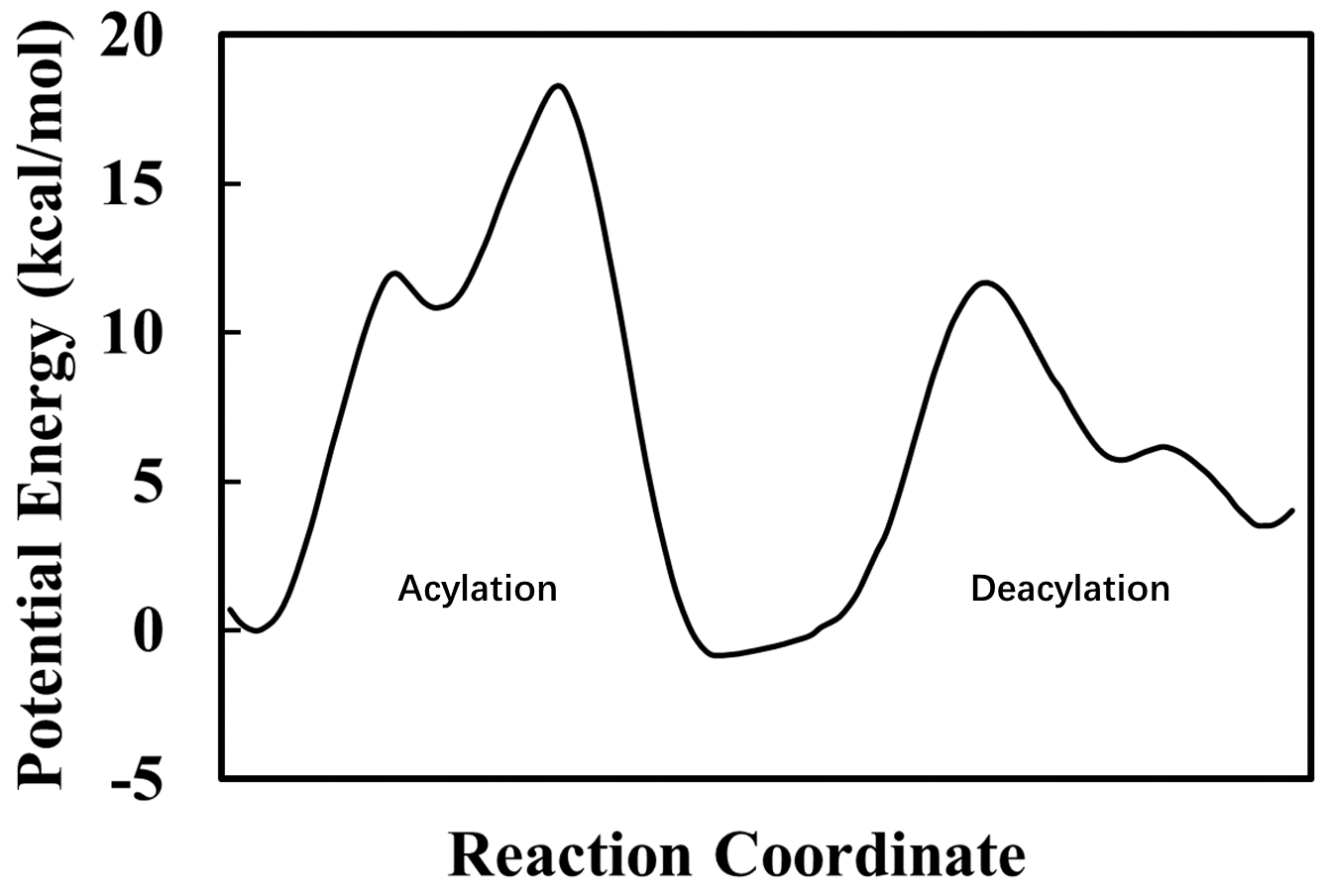


Figure S1. The potential energy profile determined by the QM/MM reaction-coordinate calculations on Kuma030-catalyzed hydrolysis of the substrate (PFPQPQQPF) at the QM/MM(DFTB3:CHARMM36) level. The methods are the same with the previous publication (JCIM, 2017**,** 57 (5), 1179-1186.). In addition, the same reaction catalyzed by KumaWT shows a similar potential energy profile with Kuma030 (data not shown).
